# Identification of differentially expressed genes and pathways between intramuscular and abdominal fat-derived preadipocyte differentiation of chickens in vitro

**DOI:** 10.1186/s12864-019-6116-0

**Published:** 2019-10-15

**Authors:** Meng Zhang, Fang Li, Xiang-fei Ma, Wen-ting Li, Rui-rui Jiang, Rui-li Han, Guo-xi Li, Yan-bin Wang, Zi-yi Li, Ya-dong Tian, Xiang-tao Kang, Gui-rong Sun

**Affiliations:** 1 College of Animal Science and Veterinary Medicine, Henan Agricultural University, Zhengzhou, 450002 China; 2 Henan Innovative Engineering Research Center of Poultry Germplasm Resource, Zhengzhou, 450002 China; 3 The First Hospital, Jilin University, Changchun, 130021 Jilin China

**Keywords:** Chickens, Abdominal fat, Intramuscular fat, Adipocyte differentiation, Transcriptome

## Abstract

**Background:**

The distribution and deposition of fat tissue in different parts of the body are the key factors affecting the carcass quality and meat flavour of chickens. Intramuscular fat (IMF) content is an important factor associated with meat quality, while abdominal fat (AbF) is regarded as one of the main factors affecting poultry slaughter efficiency. To investigate the differentially expressed genes (DEGs) and molecular regulatory mechanisms related to adipogenic differentiation between IMF- and AbF-derived preadipocytes, we analysed the mRNA expression profiles in preadipocytes (0d, Pre-) and adipocytes (10d, Ad-) from IMF and AbF of Gushi chickens.

**Results:**

AbF-derived preadipocytes exhibited a higher adipogenic differentiation ability (96.4% + 0.6) than IMF-derived preadipocytes **(**86.0% + 0.4) (*p* < 0.01**)**. By Ribo-Zero RNA sequencing, we obtained 4403 (2055 upregulated and 2348 downregulated) and 4693 (2797 upregulated and 1896 downregulated) DEGs between preadipocytes and adipocytes in the IMF and Ad groups, respectively. For IMF-derived preadipocyte differentiation, pathways related to the PPAR signalling pathway, ECM-receptor interaction and focal adhesion pathway were significantly enriched. For AbF-derived preadipocyte differentiation, the steroid biosynthesis pathways, calcium signaling pathway and ECM-receptor interaction pathway were significantly enriched. A large number of DEGs related to lipid metabolism, fatty acid metabolism and preadipocyte differentiation, such as *PPARG*, *ACSBG2*, *FABP4*, *FASN*, *APOA1* and *INSIG1*, were identified in our study.

**Conclusion:**

This study revealed large transcriptomic differences between IMF- and AbF-derived preadipocyte differentiation. A large number of DEGs and transcription factors that were closely related to fatty acid metabolism, lipid metabolism and preadipocyte differentiation were identified in the present study. Additionally, the microenvironment of IMF- and AbF-derived preadipocyte may play a significant role in adipogenic differentiation. This study provides valuable evidence to understand the molecular mechanisms underlying adipogenesis and fat deposition in chickens.

## Background

Chicken is generally accepted as one of the main protein sources worldwide. In the last several decades, meat quality has decreased as a result of genetic selection for growth rate and feed conversion. Intramuscular fat (IMF) content, an important factor influencing meat quality, contributes to multiple meat quality characteristics, such as flavour, tenderness and juiciness [1–3]. Abdominal fat (AbF) is an important carcass trait in chickens. A higher growth rate induces larger fiber diameters and lower IMF deposition, which severely deteriorates the quality of meat [4, 5]. However, the overemphasis on selection for a rapid growth rate leads to excessive fat accumulation, especially AbF accumulation [6]. Excessive fat is often discarded as waste [6–8]. Reducing the levels of AbF and increasing the levels of IMF have therefore become a major breeding goals in the chickens industry [6, 9].

Previous studies have indicated that adipose tissues from different locations display unique physiological and biochemical characteristics [10–12]. In addition, glucose utilization and lipid metabolism mechanisms and hormone sensitivities are different among tissues from different locations [13–16]. IMF has specific biological features compared with fat from other locations. Previous studies have suggested that AbF has higher triglyceride (TG) levels than IMF tissue [17–19]. Hrdinka C et al. demonstrated that the fatty acid composition of AbF differs significantly from that of IMF [20]. Zhou et al. found that dietary supplementation with 3% conjugated linoleic acid (CLA) decreased AbF accumulation but increased IMF content [21]. Leng et al. indicated that a desirable broiler line with high IMF content but low AbF content could be obtained by genetic selection [6]. This may be because AbF deposition and IMF deposition are subject to different regulatory mechanisms. However, the mechanisms underlying regional differences in chicken adipogenesis remain unknown.

In the current study, Ribo-Zero RNA-Seq was used to systematically identify differentially expressed genes (DEGs), mRNAs (DEMs) and novel genes (DENGs) and different pathways between preadipocytes and adipocytes of IMF and AbF. These data may contribute to a more thorough understanding of tissue-specific adipogenic differentiation and poultry meat quality.

## Results

### Ribo-zero RNA-Seq of different chicken different adipose tissue-derived preadipocytes and adipocytes

**Fig. 1:**
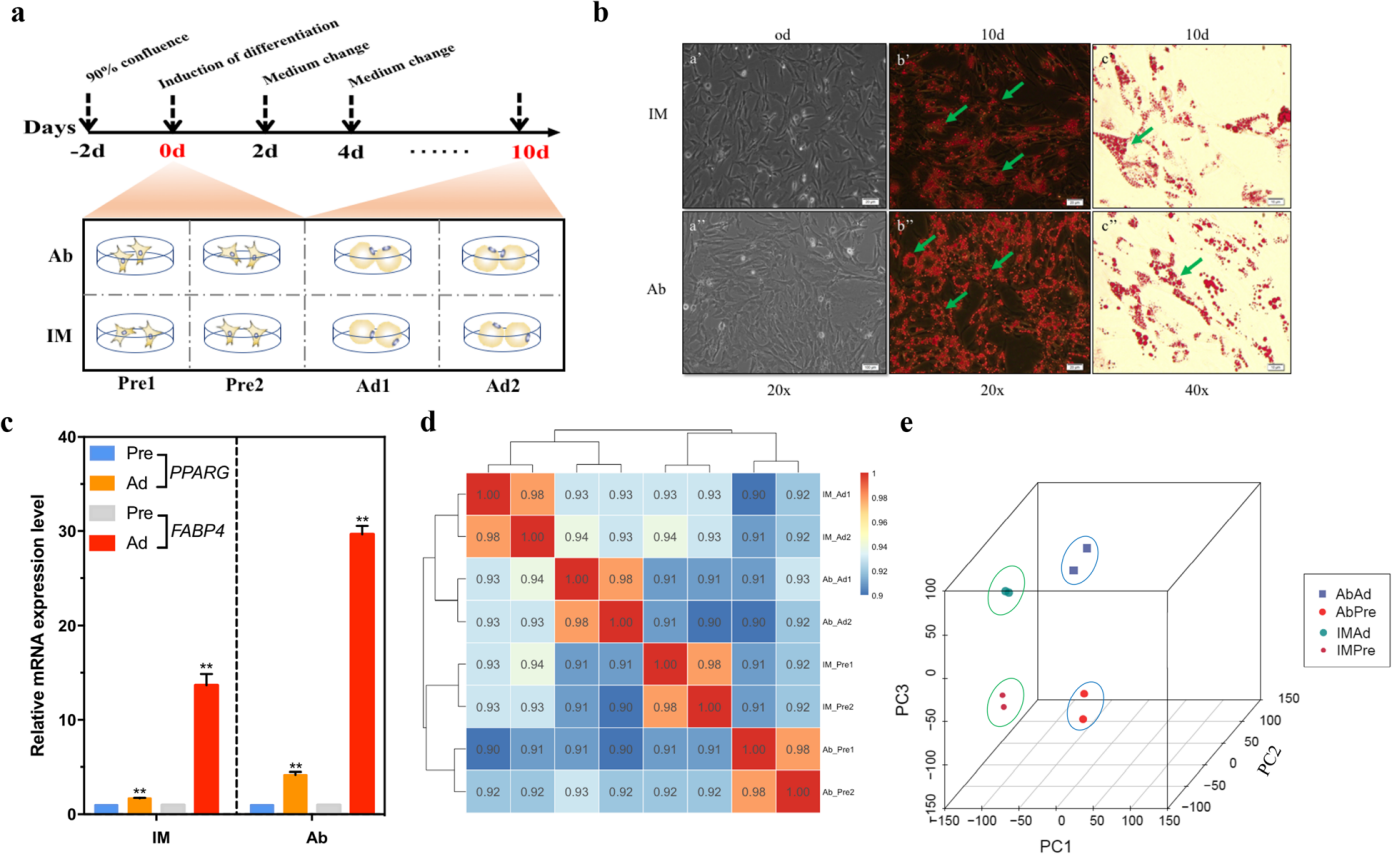
Ribo-Zero RNA-Seq of chicken preadipocytes and adipocytes. **a** Procedure for inducing the differentiation of abdominal (Ab) and intramuscular (IM) preadipocytes (upper panel). Cells were collected for RNA-Seq at day 0 (Pre) and 10 days (Ad) (lower panel). Each stage included two biological replicates. Basal medium: DMEM/F12 + 10% FBS; differentiation induction medium: basal medium + DMI + oleate; maintenance medium: basal medium + insulin. The differentiation induction medium was replaced with maintenance medium after 2 days, and the maintenance medium was in turn replaced with basal medium at day four. **b** Oil Red O staining of preadipocytes (0d) and adipocytes (10d) in the IMF and AbF groups. Arrow indicates cytosolic lipid droplets. **c** qRT-PCR analysis of the adipogenic markers *PPARG* and *FABP4* to confirm the identity of the chicken preadipocytes (mean ± SE, *n* = 3, **p ≤ 0.01). **d** Heatmap showing the results of correlation analyses between different samples. A correlation coefficient closer to 1 indicates a higher similarity between samples. **e** PCA of different samples based on the normalized expression levels of all expressed genes

Intramuscular and abdominal preadipocytes were isolated and cultured in growth medium until they reached 80–90% confluence (Fig. 1a). Microscopy showed that the IMF preadipocytes shared the same fibroblast-like morphology as the AbF preadipocytes (Fig. 1b). To construct intramuscular and abdominal adipogenic differentiation models, MDI medium supplemented with oleic acid was used for adipogenic differentiation. After induction with adipogenic agents for 10 days, chicken preadipocytes readily differentiated into mature adipocytes, and lipid droplets were visible under a microscope after 10 days of induction (Fig. 1b). AbF-derived preadipocytes exhibited a higher adipogenic differentiation ability (96.4% + 0.6) than IMF-derived preadipocytes **(**86.0% + 0.4) (*p* < 0.01**)**. The expression level of the adipogenic marker genes peroxisome proliferator-activated receptor γ (*PPARγ*, *PPARG*) and fatty acid binding protein 4 (*FABP4, ap2*) significantly increased with adipogenic differentiation (*p* < 0.01) (Fig. 1c). Pearson correlation analysis showed that the gene expression correlation coefficient within each group was noticeably higher than that between the groups, reflecting a good linear correlation between the independent samples of preadipocytes or adipocytes in the IMF and AbF groups (Pearson correlation coefficient, *r* = 0.98) (Fig. 1d). Principal component analysis (PCA) also showed global differences among the preadipocyte, adipocyte, IMF and AbF groups (Fig. 1e). All evidence suggested that our data was repeatability and reproducibility.

### Global analysis of gene expression patterns in chicken adipocytes

**Fig. 2:**
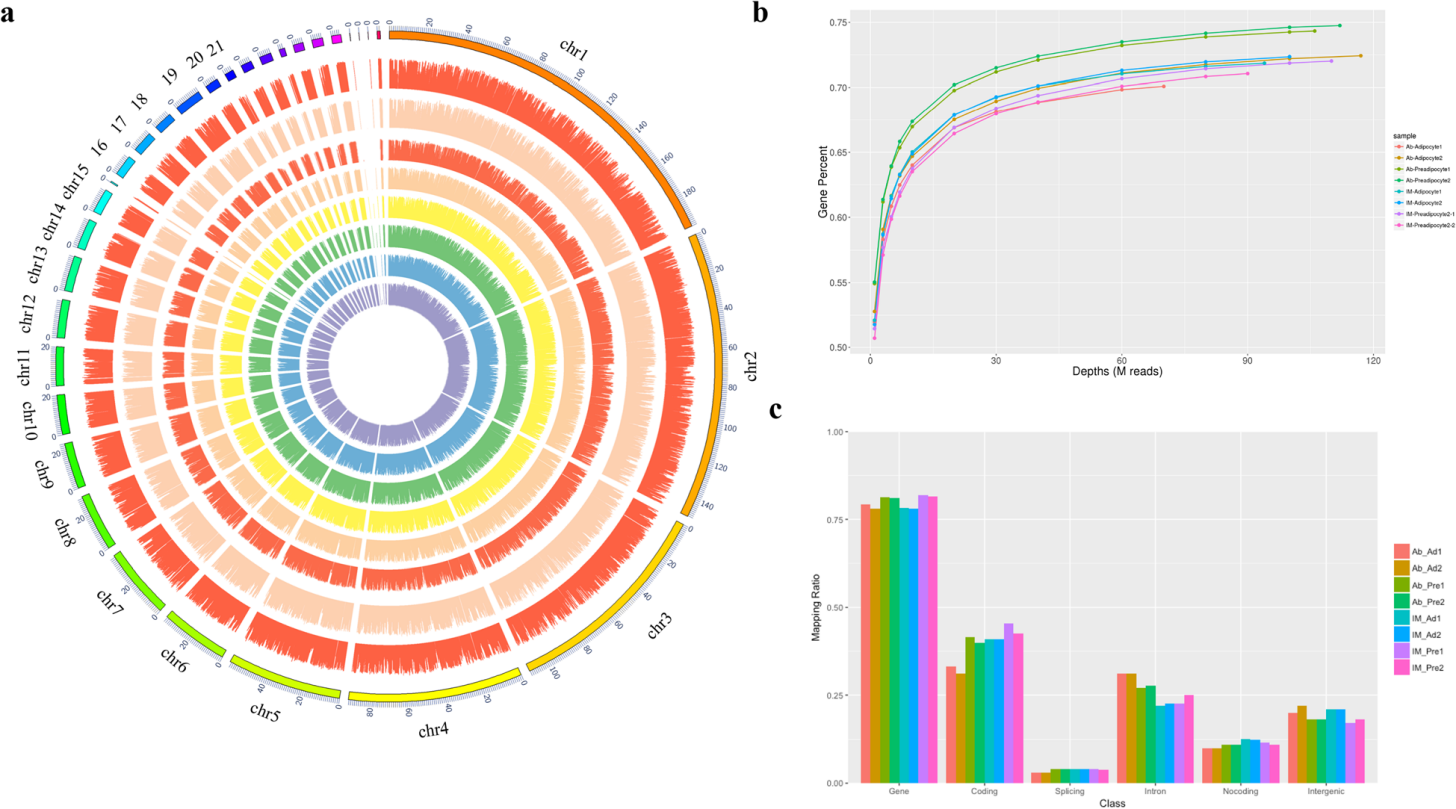
Global analysis of gene expression in chicken adipocytes. **a** Circos plot showing the distributions of the genes in the different samples on different chromosomes. From the inside circle to the outer circle: AbAd1, AbAd2, AbPre1, AbPre2, IMAd1, IMAd2, IMPre1, and IMPre2. **b** Saturation analysis of the transcriptome sequencing data from eight chicken adipocyte libraries. **c** Distribution of the mapped reads on different regions of the chicken reference genome

As shown in Table 1, 963,374,122 raw reads were produced from 8 cDNA libraries. We identified a considerable number of genes in preadipocytes and adipocytes derived from chicken breast muscle and abdominal tissues. The percentage of clean reads in each library ranged from 94.21 to 96.09%. The mapping percentage of the 8 samples ranged from 88.82 to 93.42%. We found that the genomic loci of the genes were widely distributed across chromosomes (Fig. 2a). The sequencing depth was saturated at 68 M reads for each library (Fig. 2b). The mapping percentages of the different samples on different regions of the genome are displayed in Fig. 2c. More than 75% of the reads were mapped to gene regions. In preadipocytes and adipocytes derived from breast muscle and abdominal tissues, less than 0.1% of the reads were mapped to splicing sites of the genome.

**Table 1:** Characteristics of the reads from eight chicken adipocyte libraries

Sample ID^a^	Raw reads	Clean reads	Clean ratio^b^	Mapping ratio^c^	Q20 ratio (%)
AbAd1	75,595,006	72,548,947	95.97%	92.85%	95.87
AbAd2	126,110,450	120,496,454	95.55%	93.42%	96.01
AbPre1	114,650,344	109,068,167	95.13%	92.82%	95.75
AbPre2	119,804,518	115,123,603	96.09%	92.37%	96.04
IMAd1	100,975,170	96,608,787	95.68%	89.38%	96.10
IMAd2	107,859,100	103,169,969	95.65%	88.45%	95.88
IMPre1	120,678,792	113,687,015	94.21%	92.47%	95.64
IMPre2	96,725,572	92,307,799	95.43%	88.82%	96.24

^a^AbAd and IMAd respectively represented ADF- and IMF-derived adipocyte groups; AbPre and IMPre respectively represented ADF- and IMF-derived preadipocyte groups. ^b^Clean ratio = (Clean reads/Raw reads)%; ^c^Mapping ratio = Mapped reads/All reads

### DEG, DEM and DENG profiles between different chicken adipose tissue-derived preadipocytes and adipocytes

**Fig. 3:**
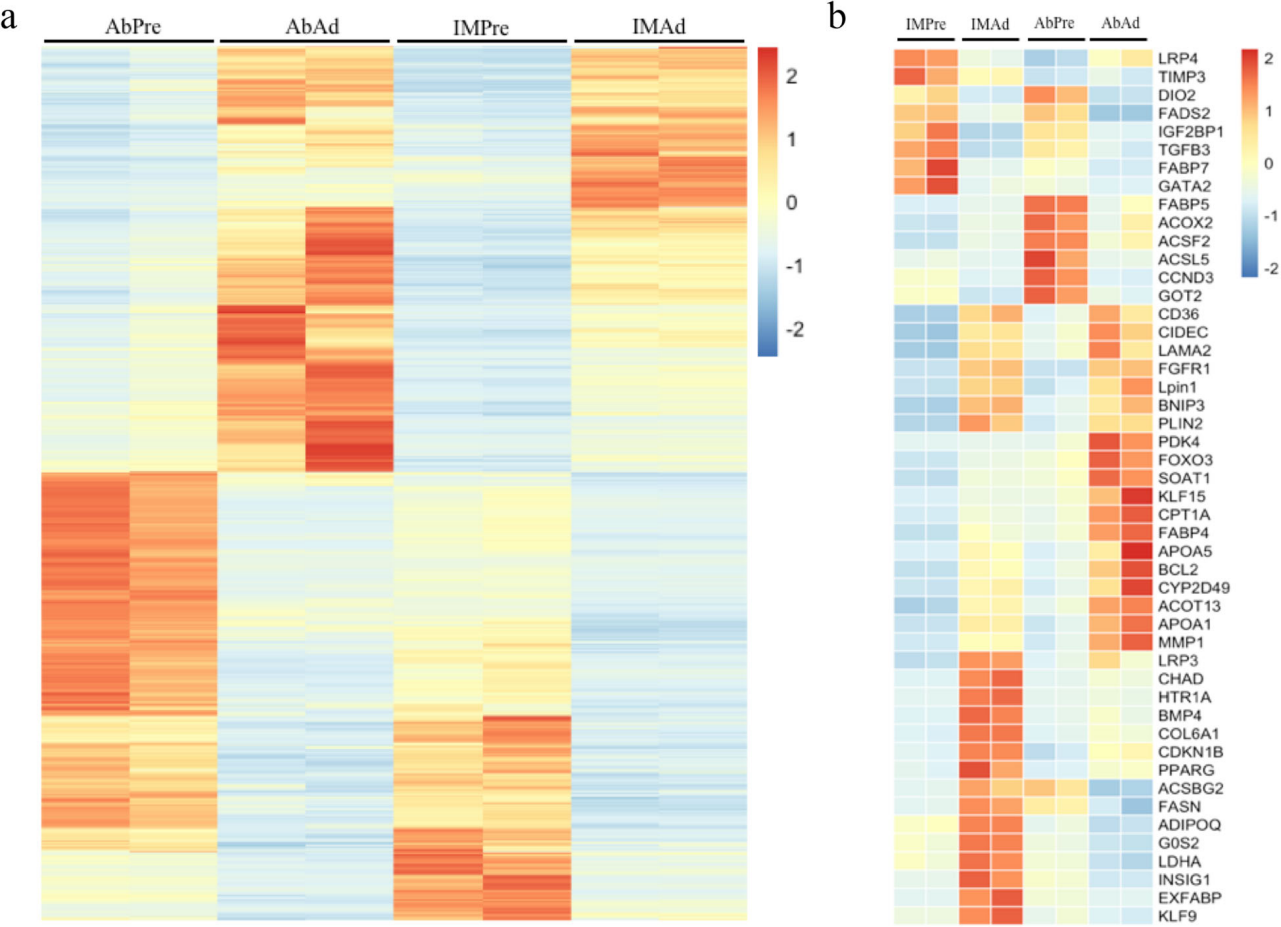
Differentially expressed genes in chicken adipocytes. **a** Heatmap of the DEGs during adipogenesis for different chicken adipose tissue-derived adipocytes. **b** Heatmap of the DEGs associated with lipid metabolism in the present study

**Fig. 4:**
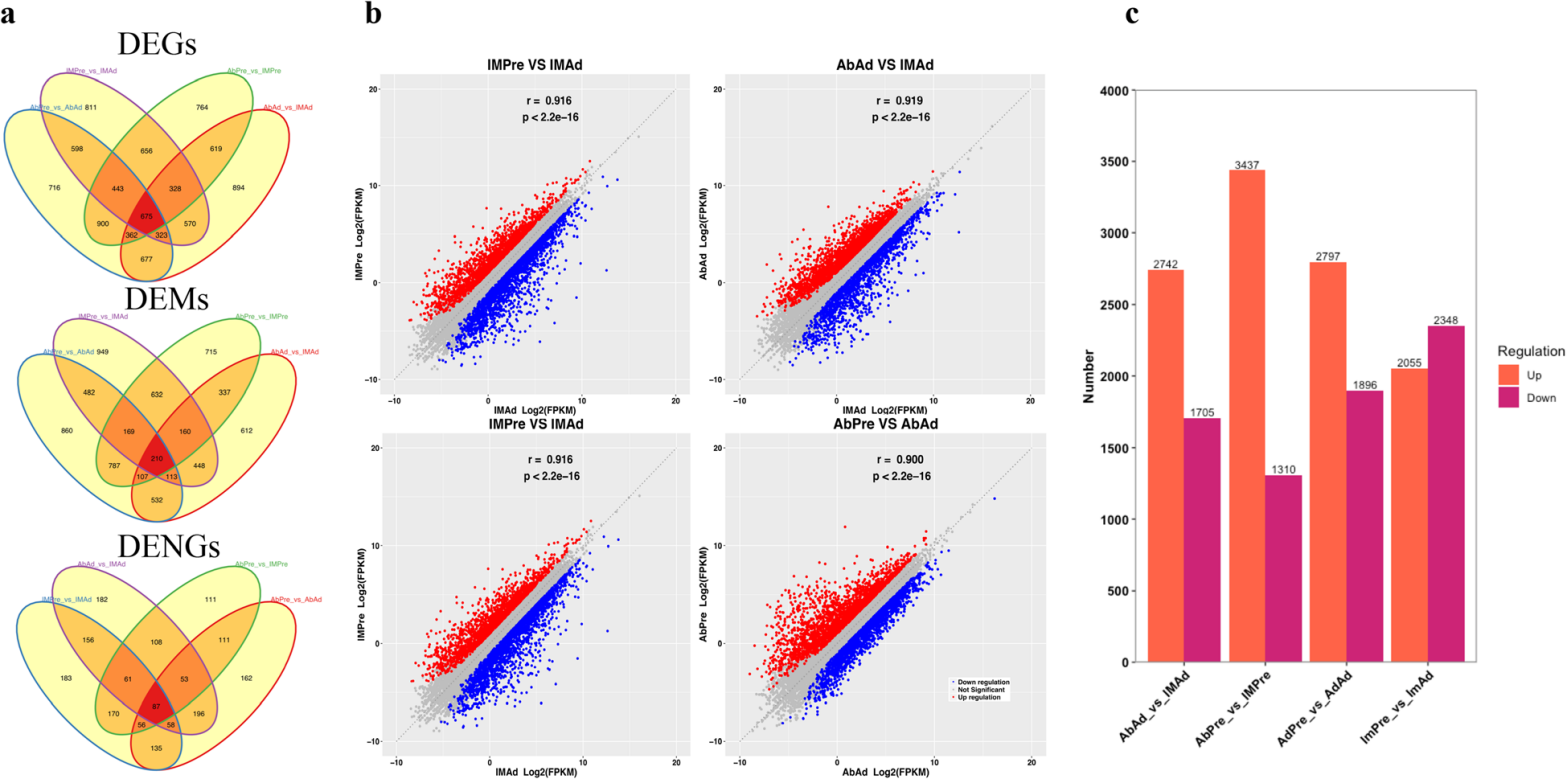
DEGs, DEMs and DENGs among four groups. **a** Venn diagram of the DEGs, DEMs and DENGs among four groups. **b** Genes expression correlation between two groups. **c** Bar plot of the DEGs that were upregulated or downregulated among four groups

To identify potential candidate genes related to adipogenic differentiation, we examined the expression level of genes in preadipocytes and adipocytes. A total of 2039 genes were found to be differentially expressed between preadipocytes and adipocytes in both the IMF and AbF groups (Fig. 3a). As expected, we noticed that large numbers of genes or transcription factors related to adipogenic differentiation and lipid metabolism were differentially expressed, including peroxisome proliferator-activated receptor γ (*PPARG),* bone morphogenetic protein 4 *(BMP4),* fatty acid synthase *(FASN),* adiponectin, C1Q and collagen domain-containing *(ADIPOQ),* perilipin 2 *(PLIN2),* lipin 1 *(LPIN1),* and carnitine palmitoyltransferase 1A *(CPT1A)* (Fig. 3b). The ten most abundant DEGs between preadipocytes and adipocytes in the IMF and AbF groups are presented in Table 2. Furthermore, we determined the global gene expression profiles in preadipocytes and adipocytes between different groups. The number of differentially expressed genes (DEGs) (Additional file 1: Table S1), mRNAs (DEMs) (Additional file 2: Table S2) and novel genes (DENGs) (Additional file 3: Table S3) between the different groups are shown in Fig. 4a. As illustrated in Fig. 4b, the expression levels of the genes of the two different samples exhibited a significant positive correlation (*R*^*2*^ > 0.81, *p* < 0.01) (Fig. 4b). We found that 2742 DEGs were upregulated and 1705 DEGs were downregulated in the AbAd group compared with the IMAd group, that 3437 DEGs were upregulated and 1310 DEGs were downregulated in the AbPre group compared with the IMPre group, that 2797 DEGs were upregulated and 1896 DEGs were downregulated in the AbPre group compared with the AbAd group, and that 2055 DEGs were upregulated and 2348 DEGs were downregulated in the IMPre group compared with the IMAd group (Fig. 4c).

**Table 2:** The top 10 most abundantly DEGs between preadipocyte and adipocyte in IMF and AbF groups

Groups	Gene ID	Gene Name	Pre	Ad	log2FC	Qvalue	Pre VS Ad
IMF	ENSGALG00000043064	*EXFABP*	2.45	6303.41	−11.33	0	DOWN
	ENSGALG00000009920	*COCH*	0.06	134.63	−11.24	0	DOWN
	ENSGALG00000030886	*PTGDS*	0.35	683.12	−10.95	0	DOWN
	ENSGALG00000007114	*APOA1*	4.00	928.17	−7.86	9.94E-310	DOWN
	ENSGALG00000008439	*CD36*	0.95	158.48	−7.38	7.80E-255	DOWN
	ENSGALG00000015090	*PLIN2*	84.52	1333.09	−3.98	1.92E-123	DOWN
	ENSGALG00000035345	*TXNRD1*	46.86	705.14	−3.91	3.05E-125	DOWN
	ENSGALG00000042388	*LAMA2*	0.11	32.04	−8.24	2.94E-265	DOWN
	ENSGALG00000015433	*ABCA1*	0.65	58.08	−6.49	4.59E-247	DOWN
	ENSGALG00000011511	*CKB*	4.91	317.33	−6.02	2.22E-207	DOWN
AbF	ENSGALG00000007114	*APOA1*	132.07	1627.15	−3.62	6.80E-06	DOWN
	ENSGALG00000009700	*PDK4*	68.41	684.73	−3.32	6.93E-05	DOWN
	ENSGALG00000030025	*FABP4*	736.89	2812.84	−1.93	6.13E-11	DOWN
	ENSGALG00000015090	*PLIN2*	296.16	1091.98	−1.88	4.11E-10	DOWN
	ENSGALG00000003580	*MMP2*	143.04	498.55	−1.80	3.33E-09	DOWN
	ENSGALG00000005974	*COL6A1*	266.56	595.62	−1.16	6.90E-05	DOWN
	ENSGALG00000009626	*THBS1*	1122.14	556.32	1.01	3.56E-02	UP
	ENSGALG00000040896	*FASN*	156.98	68.51	1.20	3.80E-04	UP
	ENSGALG00000005678	*FLNB*	537.05	141.80	1.92	4.63E-07	UP
	ENSGALG00000003578	*FN1*	984.39	254.02	1.95	5.53E-11	UP

### Functional enrichment analysis of DEGs involved in the adipogenic differentiation of chicken preadipocytes

**Fig. 5:**
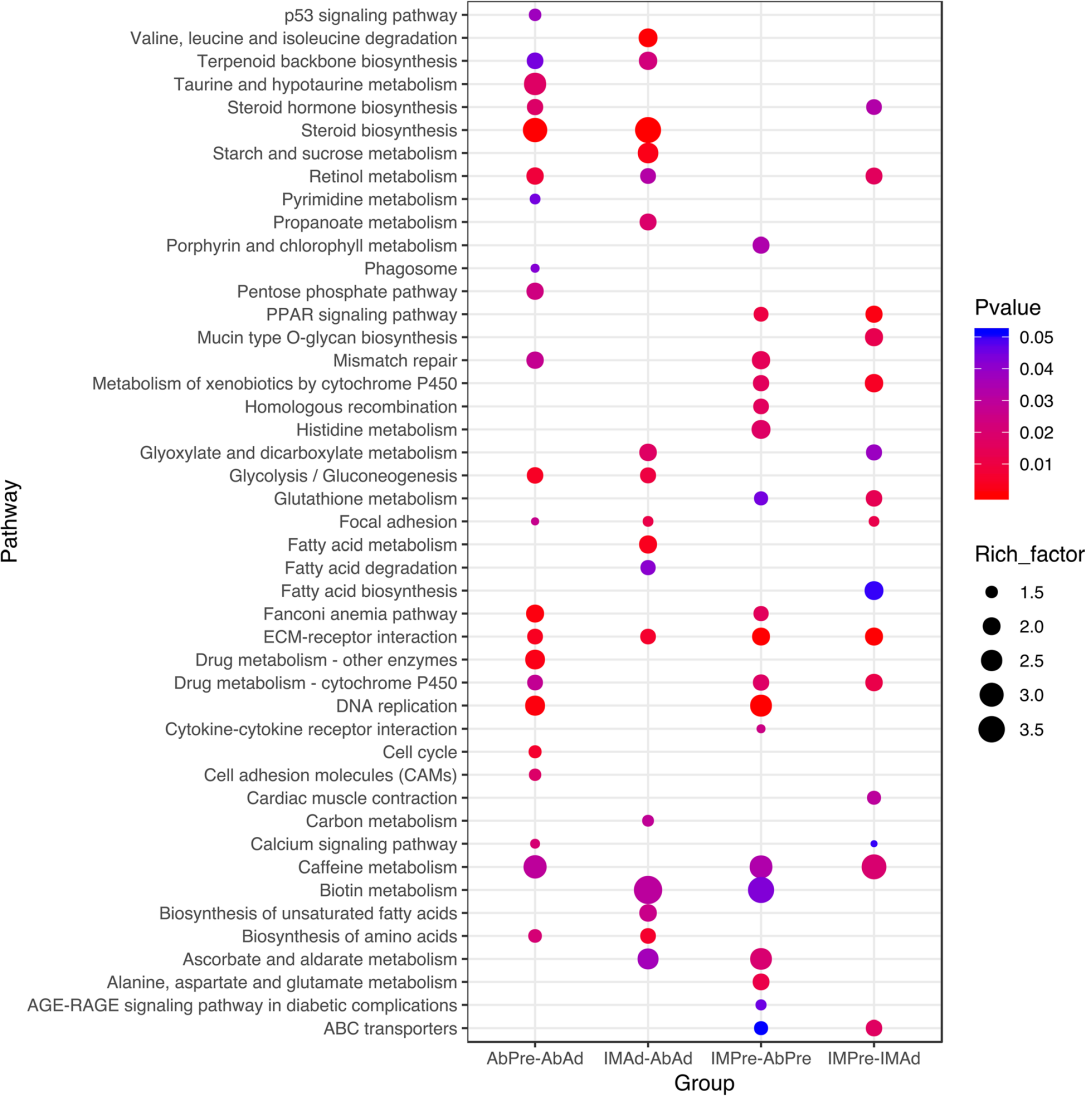
KEGG pathway enrichment analysis of the DEGs among four groups

**Fig. 6:**
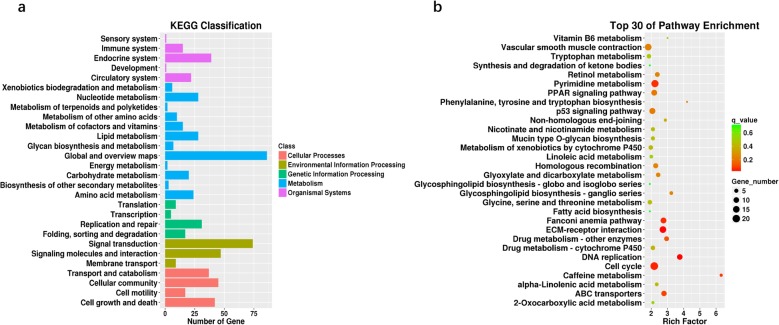
KEGG classification (**a**) and enrichment (**b**) analysis of the DEGs between AbF- and IMF-derived adipocytes during adipogenesis

**Fig. 7:**
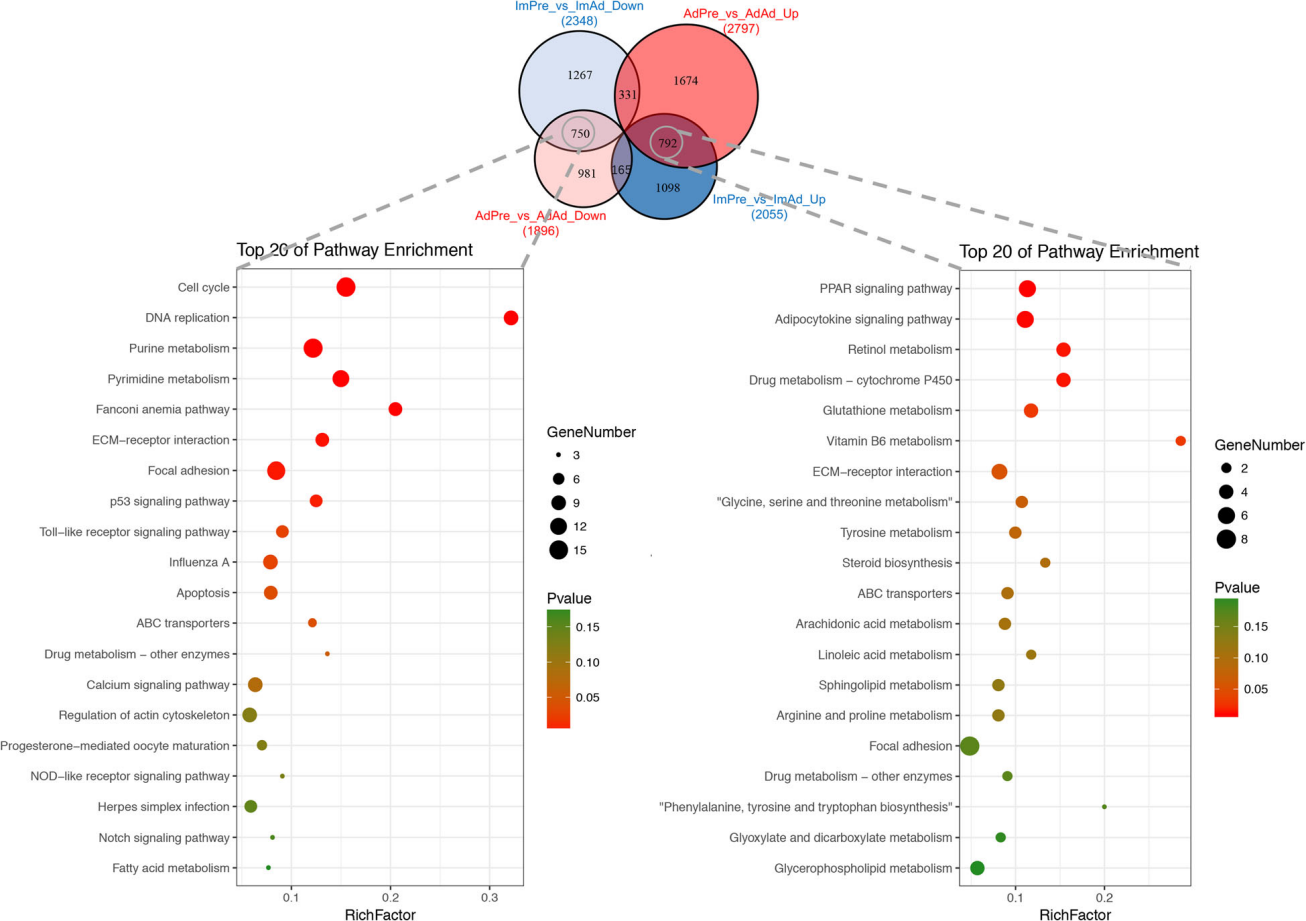
KEGG pathway enrichment analysis of the up- and downregulated DEGs shared in both the IMF and AbF groups during adipogenesis

To investigate the functions of the DEGs in chicken adipogenic differentiation, GO (Additional file 4: Table S4) and KEGG pathway (Additional file 5: Table S5) analyses were performed in the present study. Our results suggested that the DEGs in the AbPre vs AbAd and IMPre vs IMAd comparisons were significantly enriched in ECM-receptor interaction, the PPAR signalling pathway, and focal adhesion (Fig. 5). Interestingly, we found that ABC transporters, glutathione metabolism and fatty acid biosynthesis were significantly enriched for the IMF groups, while steroid biosynthesis and the p53 signalling pathway were significantly enriched for the AbF groups. The DEGs in the IMPre vs AbPre and IMAd vs AbAd comparisons were significantly enriched in the focal adhesion, ECM-receptor interaction, and fatty acid metabolism pathways, among others (Fig. 5). To identify the gene expression patterns associated with adipogenic differentiation in both IMF- and AbF-derived adipocytes, the DEGs shared between the IMF group and the AbF group were analysed in the present study. Our results suggested that the shared DEGs were enriched for the metabolism, cellular processes and translation terms (Fig. 6a) and for pathways including ECM-receptor interaction, DNA replication, the cell cycle and the PPAR signalling pathway (Fig. 6b). Furthermore, we found that the shared downregulated DEGs (number: 750) were significantly enriched in the cell cycle, focal adhesion and purine metabolism pathways, while the upregulated DEGs (number: 792) were significantly enriched in the PPAR signalling pathway, the adipocytokine signalling pathway and the retinol metabolism pathway (Fig. 7).

### Differentially expressed transcription factors and their potential interacting genes involved in adipogenic differentiation

**Fig. 8:**
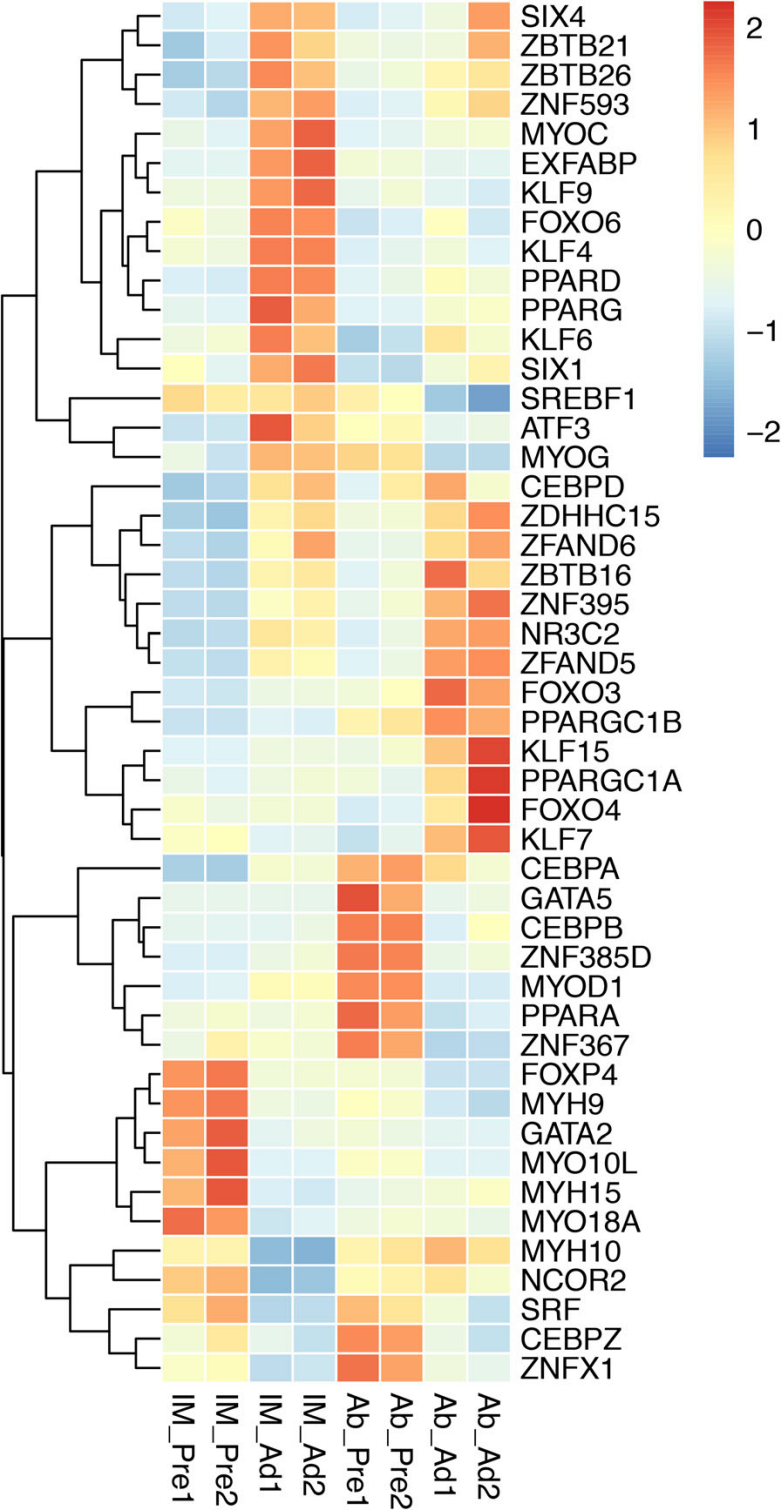
Hierarchical clustering of the transcription factors differentially expressed during adipogenesis in the IMF and AbF groups

**Fig. 9:**
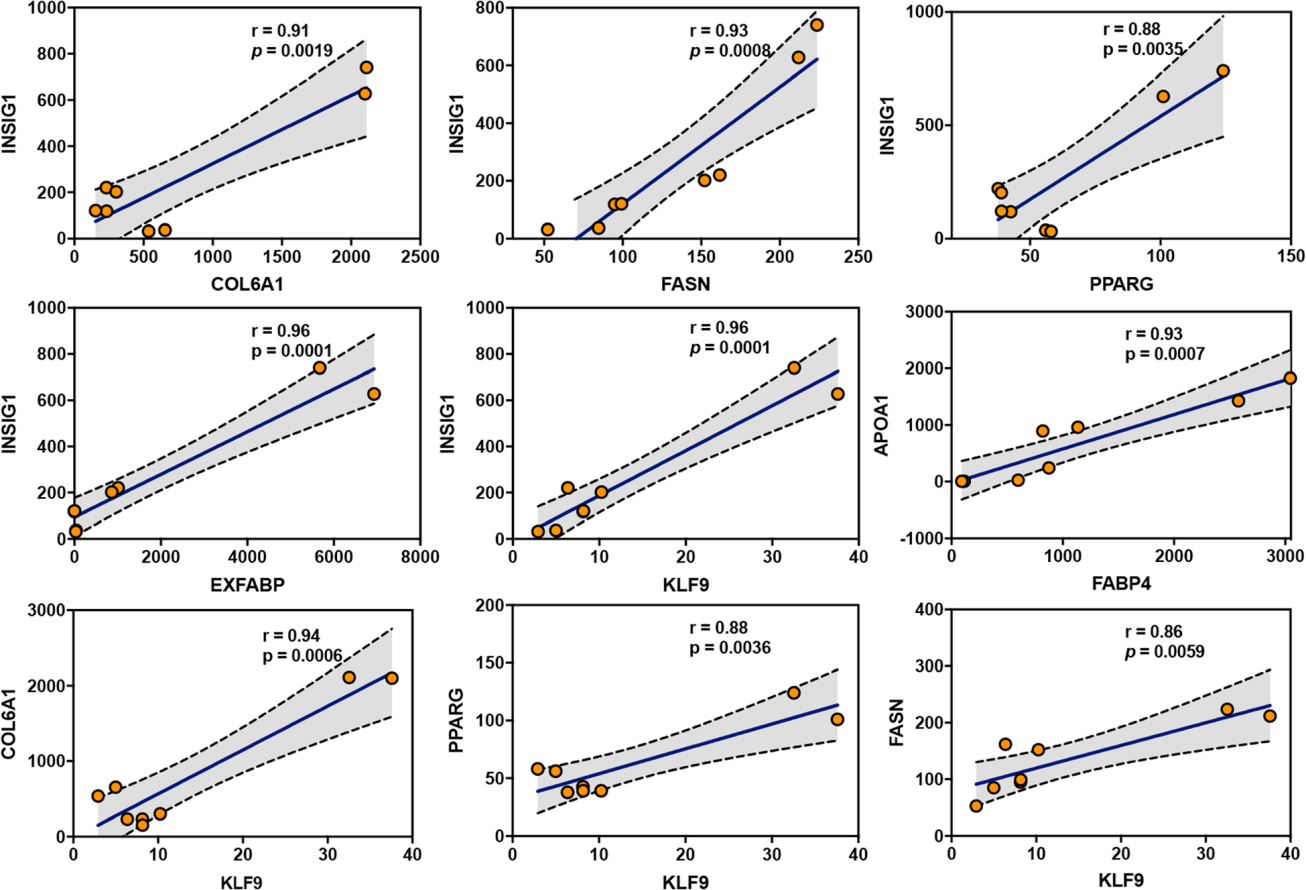
Correlation analysis of the expression levels of adipogenic differentiation-related genes

To identify the differential gene expression patterns of transcription factors in the context of adipogenic differentiation, we compared the expression levels of transcription factors (TFs). Interestingly, our results suggested that most TFs shared the same gene expression patterns in both the IMF and AbF groups, while KLF9 and MYOG showed significantly different expression patterns between the groups (Fig. 8). Furthermore, as shown in Fig. 9, the TFs and genes related to adipogenic differentiation were significantly positively correlated (r > 0.86, *p* < 0.01).

### Genes expressed in a fat depot-specific manner and the validation of the DEGs by qRT-PCR

**Fig. 10:**
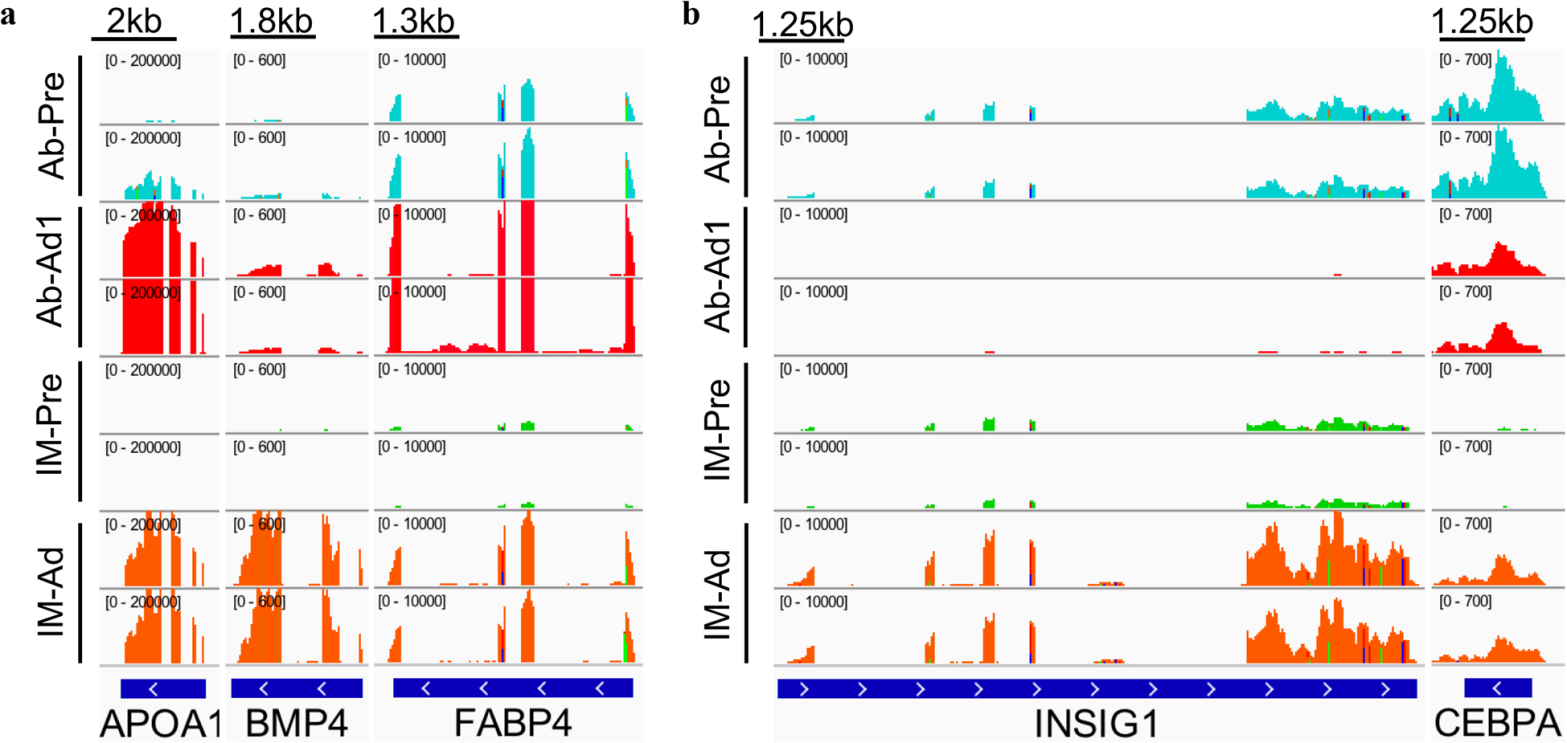
Integrative Genomics Viewer (IGV) tracks displaying the DEGs with the same (**a**) and different (**b**) gene expression patterns between the different groups

**Fig. 11:**
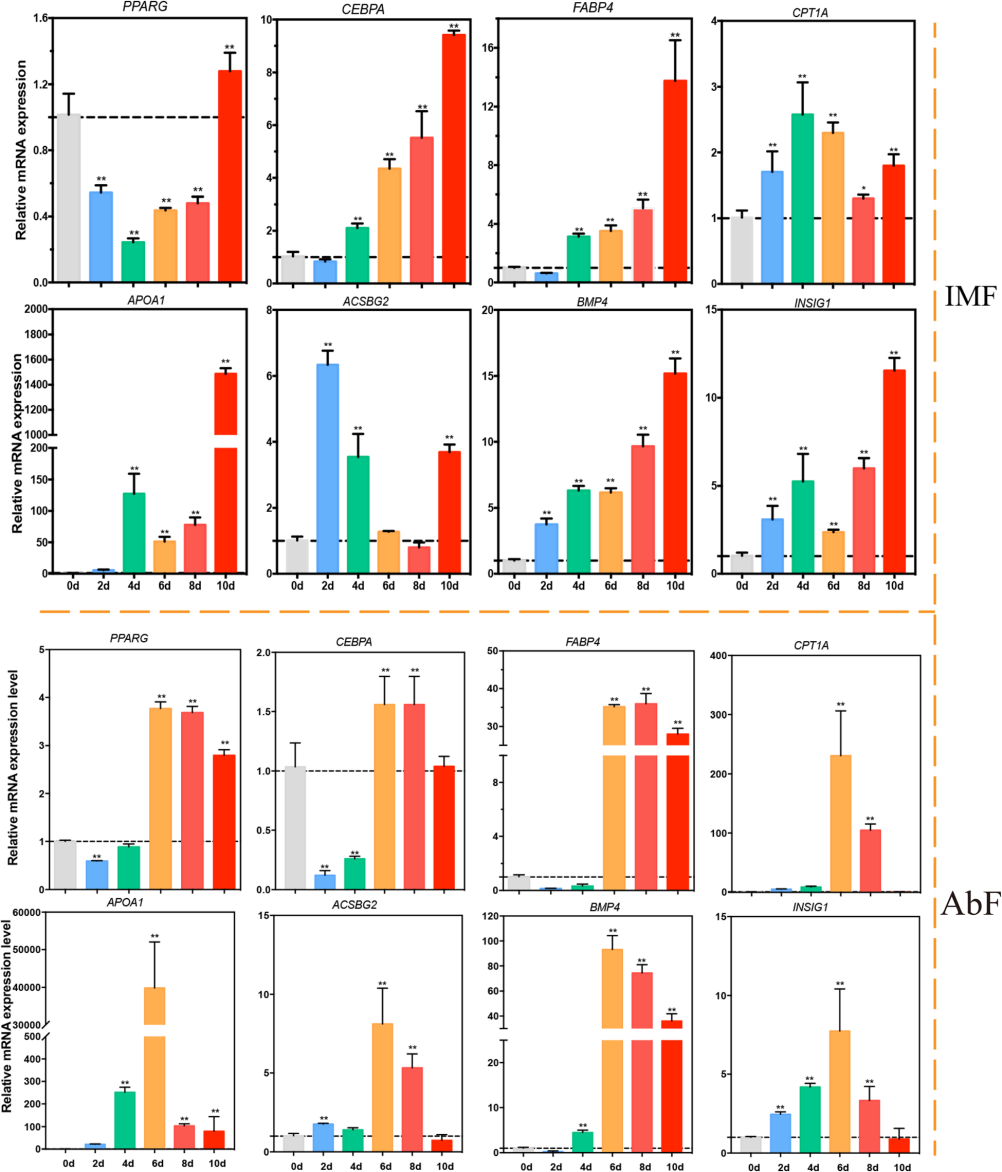
mRNA expression levels of the DEGs during adipogenesis in IMF-derived adipocytes (upper panel) and AbF-derived adipocytes (lower panel) (mean ± SE, *n* = 3, **p ≤ 0.01, *p ≤ 0.05)

To identify whether gene expression is fat depot-specific (Fig. 10), the expression levels of genes related to adipogenic differentiation at different differentiation stages and in different groups (IMF and AbF) were compared. Our results suggested that most genes have different gene expression patterns during the adipogenic differentiation process. Most genes reached the highest expression levels after 6 days of induced differentiation in the AbF group and after 8 days in the IMF group (Fig. 11). To confirm the results from RNA-Seq, qRT-PCR was performed in the present study. Our qRT-PCR results were consistent with the RNA-Seq results (Additional file 7: Figure S1).

### Integrated analysis of DEG-pathway network between different fat-derived chicken preadipocytes and adipocytes

**Fig. 12:**
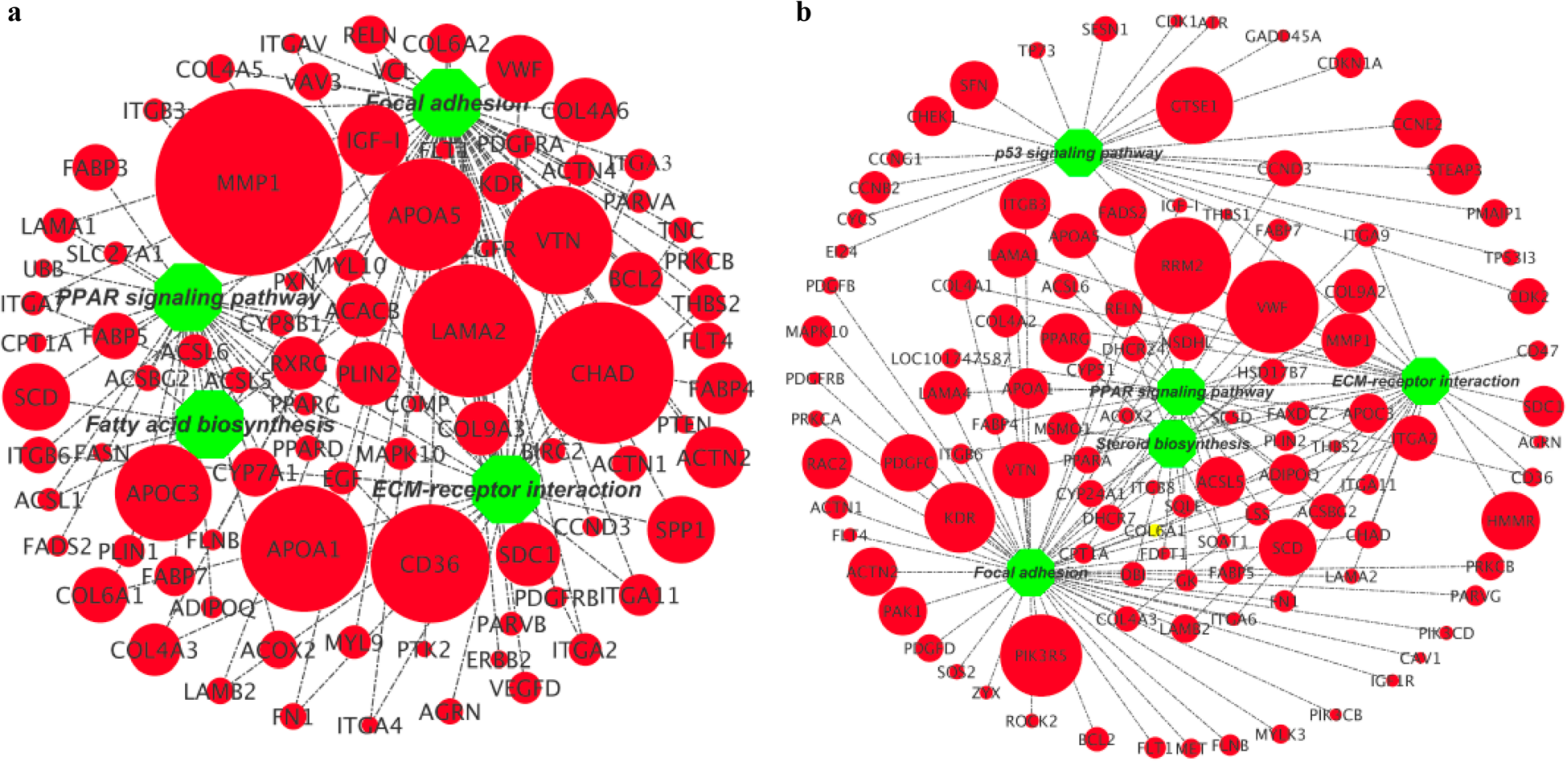
Integrated analysis of the gene-pathway networks between preadipocytes and adipocytes in the IMF (**a**) and AbF (**b**) groups

To further understand the adipogenic differentiation-regulated mechanisms of different fat-derived chicken preadipocytes, we visualized the integrated DEG-pathway networks for the IMF (Fig. 12a) and AbF (Fig. 12b) groups. Our results showed that a large number of DEGs in the IMF group were mainly enriched in the PPAR signaling pathway, fatty acid biosynthesis, focal adhesion and ECM-receptor interaction (Fig. 12a). For the AbF group, we noticed that the DEGs were mainly enriched in the PPAR signaling pathway, focal adhesion, ECM-receptor interaction, steroid biosynthesis and the p53 signaling pathway (Fig. 12b).

## Discussion

Fat deposition is mainly dependent on the proliferation, differentiation and maturation of preadipocytes [22]. IMF content is an important factor that contributes to the tenderness, juiciness and flavour of meat and thus affects meat quality. High AbF content causes low slaughter efficiency [5, 7]. It is known that IMF has high genetic correlations with abdominal fat weight (AFW) and moderate correlations with AF percentage (AFP) in chickens [23]. Previous studies have suggested that the proliferation and differentiation abilities of genes involved in lipid metabolism are dramatically lower in intramuscular adipocytes (IMAs) than in subcutaneous adipocytes (SAs) [11, 14, 24–26]. In the present study, lipids accumulated in chicken IMF and AbF preadipocytes at the late stage of differentiation. The oil red O staining results and the expression levels of two well-known adipogenic markers demonstrated that the model of adipocyte differentiation was successfully established. We noticed that the accumulation of lipids in chicken AbF adipocytes was higher than that in IMF adipocytes, consistent with the findings of previous studies in pigs [11, 27, 28].

Ribo-Zero RNA-Seq has been applied as an efficient method to explore transcriptional characteristics because it can capture both poly(A) + and poly(−) transcripts [9, 29, 30]. However, to our knowledge, none of the previous studies were carried out to compare gene expression profiles between IMF and AbF adipocytes, especially in chickens. The process by which preadipocytes differentiate into mature adipocytes is complex and is regulated by various transcription factors [31]. Previous studies have identified a large number of transcription factors, including CCAAT/enhancer-binding protein (C/EBP), peroxisome proliferator-activated receptors (PPARs) and sterol regulatory element-binding protein (SREBP) [32–35].

In this study, we detected global gene expression profiles in preadipocytes and adipocytes, providing large amounts of information for further studies on the regulatory mechanisms underlying poultry adipogenic differentiation and tissue-specific fat deposition. Our results revealed that many genes related to lipid metabolism, such as matrix metallopeptidase 2 (*MMP2)*, extracellular fatty acid-binding protein (*EXFABP)*, *CD36*, prostaglandin D2 synthase (*PTGDS)*, chondroadherin (*CHAD)*, laminin alpha 2 (*LAMA2)*, bone morphogenetic protein 4 (*BMP4)* and collagen type VI alpha 1 chain *(COL6A1),* were predominantly expressed in IMF adipocytes, whereas apolipoprotein A1 (*APOA1)*, fatty acid-binding protein 4 (*FABP4)*, perilipin 2 (*PLIN2)*, fatty acid synthetase (*FASN)*, pyruvate dehydrogenase kinase 4 (*PDK4)*, collagen type IV alpha 1 chain (*COL4*A1) and glycerol-3-phosphate dehydrogenase 1-like 2 (*GPD1L2)* were highly expressed in AbF adipocytes; these findings suggest that these genes might be involved in tissue-specific fat deposition in chickens. Previous studies have suggested that transcription factors play important roles in the regulation of adipocyte differentiation [36–39]. Transcription factors, such as Kruppel-like factors (KLFs) (*KLF9*, *KLF6* and *KLF15*), CCAAT enhancer-binding protein alpha (*CEBPA*), forkhead box O3 (*FOXO3*), myogenin (*MYOG*), sterol regulatory element-binding transcription factor 1 (*SREBF1*), nuclear receptor subfamily 3 group C member 2 (*NR3C2*), GATA-binding protein 2 (*GATA2*) and myogenic differentiation 1 (*MYOD1*), were also differentially expressed between preadipocytes and adipocytes in the different adipose tissues. KLFs and GATAs are transiently induced to control the preadipocyte-to-adipocyte transition [38, 40–42]. Furthermore, Jiang et al. suggested that NR3C1/4 may participate in intramuscular adipogenic differentiation by binding to glucocorticoid response elements in the promoters of glucocorticoid-responsive genes to activate their transcription and by regulating other transcription factors [43–45].

In the present study, functional annotation analysis of the DEGs revealed that these genes play important roles in some lipid metabolism- and adipogenic differentiation-related pathways, such as the PPAR signalling pathway, ECM-receptor interaction and fatty acid metabolism [34, 43, 46]. *PPARG*, the most adipocyte-specific and adipogenic member of the PPAR family, is mainly expressed in adipose tissue and plays an important role in lipid metabolism and adipocyte differentiation [47, 48]. The extracellular matrix (ECM) plays an important role in the regulation of proliferation, adipogenic differentiation and migration of preadipocytes [43]. Adipocyte differentiation can also be affected by fatty acid metabolism via the regulation of transcription factors [34, 49].

We noticed that *APOA1*, *CD36*, *LAMA2*, *CHAD*, *MMP1*, *RRM2*, *VWF* and *PIK3R5* were the most upregulated genes during the adipogenic differentiation processes between the IMF and AbF groups. Therefore, these genes may be involved in the positively regulating position specificity fat deposition. In addition, the differences in secretory functions and hormone sensitivities between IMF and AbF might be caused by the position-specific regulation of adipose tissue in poultry. Further studies are necessary for to elucidate the associated cell microenvironments and paracrine signaling pathways and the effects of muscle-specific regulation on adipose tissue in poultry.

## Conclusions

In conclusion, our current study showed that abdominal fat (AbF) preadipocytes accumulate more lipids than intramuscular fat (IMF) preadipocytes. This study presents the first analysis of gene expression during the differentiation of intramuscular and abdominal preadipocytes in chickens. A total of 2039 DEGs were identified by a pairwise comparison of preadipocytes at different stages of differentiation. The DEGs were found to be involved in the PPAR signalling pathway, fatty acid biosynthesis, ECM-receptor interaction and focal adhesion, consistent with previous reports on preadipocyte differentiation in chickens. These DEGs and pathways might play significant roles in intramuscular preadipocyte differentiation in chickens. Our findings provide a solid foundation for future studies on the molecular mechanisms underlying tissue-specific fat deposition and on strategies for the improvement of meat quality in poultry.

## Methods

### Ethics statement

All animal experiments were performed according to the guidelines of Henan Agricultural University (Institutional Animal Care and Use Committee (IACUC), Permit No. 11–0085, Date: 06–2011). All efforts were made to decrease animal suffering. All birds were euthanized by intraperitoneal injection of pentobarbital (Sigma, St. Louis, MO, USA) (1.0 mg/mL in methanol) at a dose of 45 mg/kg of body weight. The Gushi chickens were provided by the Animal Center of Henan Agricultural University. All birds were raised in the same environmental conditions with ad libitum water and food.

### Primary preadipocyte isolation and culture in vitro

Primary preadipocytes were isolated from the breast muscles and abdominal fat of two-week-old Gushi chickens according our previously described method [50]. In brief, breast muscle and abdominal adipose tissues were separated from the body of each chicken under sterile conditions. The tissues were washed using phosphate-buffered saline (PBS) supplemented with penicillin (100 units/mL) and streptomycin (100 μg/mL). The washed tissue was cut into 1-mm^3^ pieces and then digested with collagenase type II (1 mg/mL, Solarbio, Beijing, China) at 37 °C for 90 min. The digested cell suspension was filtered using 200- and 500-mesh screens to separate the stromal-vascular fraction from undigested tissue and mature adipocytes, and the fraction was then centrifuged at 1000 x *g* for 5 min. Preadipocytes were plated onto a 6-well culture plate at a density of 1 × 10^5^ cells/mL and maintained in Dulbecco’s modified Eagle’s medium/Ham’s nutrient mixture F-12 (DMEM/F12) supplemented with 10% FBS (Gibco, Beijing, China) with penicillin (100 units/mL) and streptomycin (100 μg/mL) in a humidified atmosphere with 5% (v/v) CO_2_ at 37 °C. For intramuscular preadipocytes, the differential adherence method was used to separate them from other cells. The basal medium was replaced with fresh medium after 2 hours.

### Preadipocyte adipogenic differentiation assay

Preadipocytes were cultured in 6-well plates until they reached 80–90% confluence. For adipogenic differentiation, upon reaching confluence (0d), the cells were exposed to differentiation medium consisting of basal medium (DMEM/F12 with 10% FBS) supplemented with 50 nM insulin, 1 μM dexamethasone, 0.5 mM 3-isobutyl-1-methylxanthine (DMI) and 300 μM oleate (dissolved in DMSO). The cells were collected at day 0, 2, 4, 6, 8, and 10 after induction. Each stage included three biological replicates (*n* = 3, three wells of cells were collected for every 2 days). The cell samples were stored at − 80 °C until use.

### RNA isolation, library preparation and sequencing

Total RNA was extracted using TRIzol reagent (TaKaRa, Dalian, China). RNA was quantified with a Qubit 2.0 (Thermo Fisher Scientific, Waltham, MA, USA) and a Nanodrop ND-2000 spectrophotometer (Thermo Fisher Scientific). Qualified total RNA was further purified with an RNA Clean XP Kit (Beckman Coulter, Inc., Kraemer Boulevard, Brea, CA, USA) and an RNase-Free DNase Set (QIAGEN, GmBH, Germany). RNA purity was assessed using an Agilent Bioanalyzer 2100 (Agilent Technologies, Santa Clara, CA, USA) with a threshold RNA integrity number > 8. The total RNA was stored at − 80 °C until use. After RNA samples were selected for library construction and deep sequencing, the rRNA was removed, and the mRNA was enriched using magnetic beads with oligo (dT) primers. RNA sequencing libraries were generated with a VAHTS™ Total RNA-seq (H/M/R) Library Prep Kit for Illumina® following the manufacturer’s instructions. The quality of all libraries was confirmed using the Agilent 2100 Bioanalyzer (Agilent Technologies, Santa Clara, CA, USA). The libraries were then analysed by using one lane of a 150 + 150-nt paired-end Illumina HiSeq 2500 run with Illumina sequencing primers. The quality of the raw data was examined using FastQC.

### Read mapping and transcriptome assembly

The splice-mapping algorithm of HISAT2 (2.0.4) [51] was used to perform genome mapping of the pre-processed reads. The clean data were mapped to the *Gallus gallus* reference genome (GGA5) with HISAT2 (2.0.4), and the default parameters were used.

### Analysis of differential gene expression patterns

To determine the differentially expressed genes, Cuffdiff [52] was used to calculate the expected number of Fragments Per Kilobase of exon model per Million mapped reads (FPKM) for each gene. StringTie (1.3.0) [53, 54] was used for quantitative analysis of the transcripts to obtain the count numbers and FPKM values of the transcripts in each sample. EdgeR [55] was used for differential gene or mRNAs analysis between samples. For biological replicates, transcripts or genes with a Qvalue (adjusted *p* value) < 0.05 and a |fold change| ≥ 2 were defined as differentially expressed genes (DEGs) or mRNAs (DEMs) between the two groups.

### Functional enrichment analysis of the differentially expressed genes (DEGs)

Gene Ontology (GO) enrichment analysis was performed with the DAVID database (https://david.ncifcrf.gov/). For Kyoto Encyclopedia of Genes and Genomes (KEGG) analysis, the differentially expressed genes or transcripts were analyzed on the KEGG online website (http://www.genome.jp/kegg/). The gene-pathway interaction networks for the DEGs were visualized with Cytoscape 3.4.0 (http://www.cytoscape.org/) [56].

### Oil red O staining

An Oil Red O staining assay was performed according to the methods in our previous study [50]. Briefly, adipocytes were gently washed with cold PBS three times and fixed with 4% paraformaldehyde for 30 min. Then, the fixed cells were gently washed with cold PBS three times, incubated with 60% filtered Oil Red O solution for 40 min and then observed under a phase-contrast microscope to check for positive cells appearing red. The cells were then washed three times with deionized cold PBS and photographed using an Olympus CKX41-F32FL microscope (Olympus, Tokyo, Japan). Subsequently, Oil Red O was eluted from the stained cells with 100% isopropanol (v/v) and quantified with a microplate reader (Thermo Fisher Scientific) at 500 nm. ImageJ software (National Institutes of Health, Bethesda, MD) was used to estimate the adipogenic differentiation ability. The cells with lipid droplets were regarded as differentiated cells: Adipogenic differentiation ratio = (differentiated cells count / total cells count) × 100%.

### RNA isolation and real-time quantitative PCR (qRT-PCR)

Primers for the DEGs were designed using Primer3Plus online software (http://www.primer3plus.com/cgi-bin/dev/primer3plus.cgi) (Additional file 6: Table S6). qPCR was performed using SYBR® Green PCR Master Mix (TaKaRa, Dalian, China). The PCR mixture contained 5 μL of SYBR® Premix Ex Taq II (2×), 0.5 μL of forward primer (10 μM), 0.5 μL of reverse primer (10 μM), and 200 ng of cDNA, with RNA-free water added to 10 μL. The qRT-PCR was conducted in a LightCycler 96 system (Roche) by the SYBR Green method. The program included an initial step of 95 °C for 5 min; 40 cycles of 95 °C for 30 s, 60 °C for 30 s, and 72 °C for 20 s; and melting curve generation, which was performed as follows: 95 °C for 10 s, annealing at 65 °C for 20 s, and heating through a continuous temperature gradient to 97 °C with 5 acquisitions/s. Chicken *GAPDH* was selected as an internal control gene. All samples were examined in triplicate. All data were analysed using the 2^-ΔΔCt^ method. All data are shown as fold changes in gene expression compared with the gene expression in the 0d group.

### Statistical analysis

Statistical significance between two experimental groups was evaluated with a T-test for comparisons in SPSS 20.0 statistical software (IBM, Chicago, IL, USA). Statistical significance among three or more experimental groups was evaluated by one-way ANOVA followed by Dunnett’s test for multiple comparisons in SPSS 22.0. Graphics were drawn using GraphPad Prism 7 (GraphPad Software, San Diego, CA, USA) and RStudio 1.1.453 software. All data are expressed as the mean ± standard error (SE). A *P*-value **≤**0.05 was considered statistically significant, and a *P*-value **≤**0.01 was considered extreme significant.

## Supplementary information

**Additional file 1: Table S1.** DEGs among four groups

**Additional file 2: Table S2.** DEMs among four groups

**Additional file 3: Table S3.** DENGs among four groups

**Additional file 4: Table S4.** GO analysis associated with DEGs among four

**Additional file 5: Table S5.** KEGG Pathways associated with DEGs among four

**Additional file 6: Table S6.** Primers used for qRT-PCR in this study

**Additional file 7: Figure S1.** The correlation analysis between RNA-Seq data and qRT-PCR results

## Data Availability

The full data sets have been submitted to NCBI with bioproject asscession number: PRJNA429489 and Sequence Read Archive (SRA) accession number: SRR6459505, SRR6459506, SRR6459503, SRR6459504, SRR6459509, SRR6459510, SRR6459507 and SRR6459508. The datasets supporting the conclusions of this article are included within the article and its additional files.

## Abbreviations

AbF: Abdominal fat
APOA1: Apolipoprotein A1
BMP4: Bone morphogenetic protein 4
CD36: CD36 molecule
CEBPA: CCAAT enhancer binding protein alpha
CEBPA: CCAAT/enhancer-binding protein alpha
CHAD: Chondroadherin
COL4A1: Collagen type IV alpha 1 chain
COL6A1: Collagen type VI alpha 1 chain
DEGs: Differentially expressed genes
DEMs: Differentially expressed mRNAs
DENGs: Differentially expressed novel genes
DMEM: Dubecco’s modified Eagle medium
EXFABP: Extracellular fatty acid-binding protein
FABP4: Fatty acid binding protein 4
FASN: Fatty acid synthetase
FOXO3: Forkhead box O3
GATA2: GATA binding protein 2
GO: Gene Ontology
GPD1L2: Glycerol-3-phosphate dehydrogenase 1-like 2
IMF: Intramuscular fat
INSIG1/2: Insulin induced gene 1/2
KEGG: Kyoto Encyclopedia of Genes and Genomes
Kruppel-like factors: (KLFs) (KLF9, KLF6 and KLF15)
LAMA2: Laminin, alpha 2
MMP1: Matrix metallopeptidase 1
MYOD1: Myogenic differentiation 1
MYOG: Myogenin
NR3C2: Nuclear receptor subfamily 3 group C member 2
PDK4: Pyruvate dehydrogenase kinase 4
PLIN2: perilipin 2
PPARs: Peroxisome proliferator-activated receptors
PTGDS: Prostaglandin D2 synthase
qRT-PCR: Quantitative real time PCR
SREBF1: Sterol regulatory element binding transcription factor 1
SREBP: Sterol regulatory element-binding protein
TG: Triglyceride

## References

[1] Nishimura T, Hattori A, Takahashi K (1999). Structural changes in intramuscular connective tissue during the fattening of Japanese black cattle: effect of marbling on beef tenderization. J Anim Sci.

[2] Chartrin P, Méteau K, Juin H, Bernadet MD, Guy G, Larzul C, Rémignon H, Mourot J, Duclos MJ, Baéza E (2006). Effects of intramuscular fat levels on sensory characteristics of duck breast meat. J Poult Sci.

[3] Fernandez X, ., Monin G, ., Talmant A, ., Mourot J, ., Lebret B: Influence of intramuscular fat content on the quality of pig meat - 2. Consumer acceptability of m longissimus lumborum. J Meat Sci 1999, 53(1):67–72. 10.1016/s0309-1740(99)00038-8 22062934

[4] Dransfield E, Sosnicki AA (1999). Relationship between muscle growth and poultry meat quality. J Poult Sci.

[5] Ismail I, Joo S (2017). Poultry Meat Quality in Relation to Muscle Growth and Muscle Fiber Characteristics. Korean J Food Sci Anim Resour.

[6] Leng L, Zhang H, Dong JQ, Wang ZP, Zhang XY, Wang SZ, Cao ZP, Li YM, Li H (2016). Selection against abdominal fat percentage may increase intramuscular fat content in broilers. J Anim Breed Genet.

[7] Emmerson DA (1997). Commercial approaches to genetic selection for growth and feed conversion in domestic poultry. Poult Sci.

[8] Jiang M, Fan WL, Xing SY, Wang J, Li P, Liu RR, Li QH, Zheng MQ, Cui HX, Wen JJ (2017). Effects of balanced selection for intramuscular fat and abdominal fat percentage and estimates of genetic parameters. Poult Sci.

[9] Qiu F, Xie L, Ma JE, Luo W, Zhang L, Chao Z, Chen S, Nie Q, Lin Z, Zhang XJ (2017). Lower Expression of SLC27A1 Enhances Intramuscular Fat Deposition in Chicken via Down-Regulated Fatty Acid Oxidation Mediated by CPT1A. Front Physiol.

[10] Sun WX, Wang HH, Jiang BC, Zhao YY, Xie ZR, Xiong K, Chen J (2013). Global comparison of gene expression between subcutaneous and intramuscular adipose tissue of mature Erhualian pig. Genet Mol Res.

[11] Zhou G, Wang S, Wang Z, Zhu X, Shu G, Liao W, Yu K, Gao P, Xi Q, Wang XJ (2010). Global comparison of gene expression profiles between intramuscular and subcutaneous adipocytes of neonatal landrace pig using microarray. Meat Sci.

[12] Cui H, Zheng M, Zhao G, Liu R, Wen J (2018). Identification of differentially expressed genes and pathways for intramuscular fat metabolism between breast and thigh tissues of chickens. BMC Genomics.

[13] Smith SB, Crouse JD (1984). Relative contributions of acetate, lactate and glucose to lipogenesis in bovine intramuscular and subcutaneous adipose tissue. J Nutr.

[14] Chu W, Wei W, Han H, Gao Y, Liu K, Tian Y, Jiang Z, Zhang L, Chen J (2017). Muscle-specific downregulation of GR levels inhibits adipogenesis in porcine intramuscular adipocyte tissue. Sci Rep.

[15] Songbo W, Guixuan Z, Gang S, Lina W, Xiaotong Z, Ping G, Qianyun X, Yongliang Z, Li Y, Qingyan J (2013). Glucose utilization, lipid metabolism and BMP-Smad signaling pathway of porcine intramuscular preadipocytes compared with subcutaneous preadipocytes. Cell Physiol Biochem.

[16] Han H, Wei W, Chu W, Liu K, Tian Y, Jiang Z, Chen J (2017). Muscle Conditional Medium Reduces Intramuscular Adipocyte Differentiation and Lipid Accumulation through Regulating Insulin Signaling. Int J Mol Sci.

[17] Hood RL, Allen CE (1973). Cellularity of bovine adipose tissue. J Lipid Res.

[18] Hood RL, Allen CE (1978). Lipogenesis in isolated intramuscular adipose tissue from four bovine muscles. J Anim Sci.

[19] Lu L, Cui H, Zheng M, Zhao G, Jie WJ (2018). Comparative analysis of differentially expressed genes related to triglyceride metabolism between intramuscular fat and abdominal fat in broilers. Br Poult Sci.

[20] Hrdinka C, Zollitsch W, Knaus W, Lettner F (1996). Effects of dietary fatty acid pattern on melting point and composition of adipose tissues and intramuscular fat of broiler carcasses. J Poult Sci.

[21] Zhou J, Shi SY, Wang JH, Pan L, Ke-Zong QI JCJoVS: Effects of conjugated linoleic acid on different adipose tissue deposits and immune function of yellow broiler chickens. 2008.

[22] Cristancho AG, Lazar MA (2011). Forming functional fat: a growing understanding of adipocyte differentiation. Nat Rev Mol Cell Biol.

[23] Chen JL, Zhao GP, Zheng MQ, Wen J, Yang N (2008). Estimation of genetic parameters for contents of intramuscular fat and inosine-5′-monophosphate and carcass traits in Chinese Beijing-You chickens. Poult Sci.

[24] Gardan D, Gondret F, Louveau I (2006). Lipid metabolism and secretory function of porcine intramuscular adipocytes compared with subcutaneous and perirenal adipocytes. Am J Physiol Endocrinol Metab.

[25] Gondret F, Guitton N, Guillerm-Regost C, Louveau I (2008). Regional differences in porcine adipocytes isolated from skeletal muscle and adipose tissues as identified by a proteomic approach. J Anim Sci.

[26] Hua ZG, Xiong LJ, Yan C, Qing ZY, Hui GP, Tian YJ, Xin ZR (2014). Comparison of the adipogenesis in intramuscular and subcutaneous adipocytes from Bamei and landrace pigs. Biochemistry and cell biology = Biochimie et biologie cellulaire.

[27] Maryline K, Pierre S (2011). A review of the factors influencing the development of intermuscular adipose tissue in the growing pig. Meat Sci.

[28] Kouba M, Bonneau M (2009). Compared development of intermuscular and subcutaneous fat in carcass and primal cuts of growing pigs from 30 to 140kg body weight. Meat Sci.

[29] Sun X, Li M, Sun Y, Cai H, Li R, Wei X, Lan X, Huang Y, Lei C, Chen H (2015). The developmental transcriptome landscape of bovine skeletal muscle defined by Ribo-zero ribonucleic acid sequencing. J Anim Sci.

[30] Zhao W, He X, Hoadley KA, Parker JS, Hayes DN, Perou CM (2014). Comparison of RNA-Seq by poly (a) capture, ribosomal RNA depletion, and DNA microarray for expression profiling. BMC Genomics.

[31] Lefterova M, Lazar M (2009). New developments in adipogenesis. Trends Endocrinol Metab.

[32] Rosen ED, Hsu CH, Wang X, Sakai S, Freeman MW, Gonzalez FJ, Spiegelman BM (2002). C/EBPα induces adipogenesis through PPARγ: a unified pathway. Genes Dev.

[33] Frédéric P, Martin K, Namjin C, Acharawan TN, Thanaset S, Rita MDO, Mark L, Mcburney MW, Leonard G (2004). Sirt1 promotes fat mobilization in white adipocytes by repressing PPAR-gamma. Nature.

[34] Kim JB, Spiegelman BM (1996). ADD1/SREBP1 promotes adipocyte differentiation and gene expression linked to fatty acid metabolism. Genes Dev.

[35] Cowherd RM, Lyle RE, Jr MG (1999). Molecular regulation of adipocyte differentiation ☆. Semin Cell Dev Biol.

[36] Nakae J, Kitamura T, Kitamura Y, Arden KC, Accili DJ (2003). The forkhead transcription factor Foxo1 regulates adipocyte differentiation. Dev Cell.

[37] Tontonoz P, Kim JB, Graves RA, Spiegelman BMJM, Biology C (1993). ADD1: a novel helix-loop-helix transcription factor associated with adipocyte determination and differentiation. Mol Cell Biol.

[38] Tong Q, Dalgin G, Xu H, Ting CN, Leiden JM, Hotamisligil GS (2000). Function of GATA Transcription Factors in Preadipocyte-Adipocyte Transition. Science.

[39] Tong Q, Tsai J, Tan G, Dalgin G, Hotamisligil GS (2005). Interaction between GATA and the C/EBP family of transcription factors is critical in GATA-mediated suppression of adipocyte differentiation. Mol Cell Biol.

[40] Jiang S, Wei H, Song T, Yang Y, Zhang F, Zhou Y, Peng J, Jiang SJ (2015). KLF13 promotes porcine adipocyte differentiation through PPARγ activation. Cell Biosci.

[41] Yamamoto K, Sakaguchi M, Medina RJ, Niida A, Sakaguchi Y, Miyazaki M, Kataoka K, Huh NH (2010). Transcriptional regulation of a brown adipocyte-specific gene, UCP1, by KLF11 and KLF15. Biochem Biophys Res Commun.

[42] Pei H, Yao Y, Yang Y, Liao K, J-R WJ (2011). Krüppel-like factor KLF9 regulates PPARγ transactivation at the middle stage of adipogenesis. Cell Death Differ.

[43] Shuzhong J, Hongkui W, Tongxing S, Yang Y, Jian P, Siwen J (2013). Transcriptome comparison between porcine subcutaneous and intramuscular stromal vascular cells during adipogenic differentiation. PLoS One.

[44] Mi-Jeong L, Da-Wei G, Burkey BF, Fried SK (2011). Pathways regulated by glucocorticoids in omental and subcutaneous human adipose tissues: a microarray study. Am J Physiol Endocrinol Metab.

[45] Chi-Yi Y, Oleg M, Lee JV, Joanna T, Charlie H, Speed TP, Jen-Chywan WJ (2010). Genome-wide analysis of glucocorticoid receptor binding regions in adipocytes reveal gene network involved in triglyceride homeostasis. PLoS One.

[46] Cai H, Li M, Sun X, Plath M, Li C, Lan X, Lei C, Huang Y, Bai Y, Qi X (2018). Global Transcriptome Analysis During Adipogenic Differentiation and Involvement of Transthyretin Gene in Adipogenesis in Cattle. Front Genet.

[47] Michael L, Lazar MA (2005). The many faces of PPARgamma. Cell.

[48] Wang Y, Mu Y, Li H, Ding N, Wang Q, Wang Y, Wang S, Wang N (2008). Peroxisome proliferator-activated receptor-gamma gene: a key regulator of adipocyte differentiation in chickens. Poult Sci.

[49] Schaffer JE, Lodish HF (1994). Expression cloning and characterization of a novel adipocyte long chain fatty acid transport protein. Cell.

[50] Zhang M, Li DH, Li F, Sun JW, Jiang RR, Li ZJ, Han RL, Li GX, Liu XJ, Kang XT (2018). Integrated analysis of MiRNA and genes associated with meat quality reveals that Gga-MiR-140-5p affects intramuscular fat deposition in chickens. Cell Physiol Biochem.

[51] Kim D, Langmead B, Salzberg SL (2015). HISAT: a fast spliced aligner with low memory requirements. Nat Methods.

[52] Trapnell C, Hendrickson DG, Sauvageau M, Goff L, Rinn JL, Pachter L (2013). Differential analysis of gene regulation at transcript resolution with RNA-seq. Nat Biotechnol.

[53] Pertea M, Pertea GM, Antonescu CM, Chang TC, Mendell JT, Salzberg SL (2015). StringTie enables improved reconstruction of a transcriptome from RNA-seq reads. Nat Biotechnol.

[54] Pertea M, Kim D, Pertea GM, Leek JT, Salzberg SL (2016). Transcript-level expression analysis of RNA-seq experiments with HISAT, StringTie and Ballgown. Nat Protoc.

[55] Robinson MD, DJ MC, Smyth GK (2010). edgeR: a Bioconductor package for differential expression analysis of digital gene expression data. Bioinformatics (Oxford, England).

[56] Shannon P, Markiel A, Ozier O, Baliga NS, Wang JT, Ramage D, Amin N, Schwikowski B, Ideker T (2003). Cytoscape: a software environment for integrated models of biomolecular interaction networks. Genome Res.

