# Supplementary figures and images for: Identification of differentially expressed genes and pathways between intramuscular and abdominal fat-derived preadipocyte differentiation of chickens in vitro
Source: BMC Genomics. 2019 Oct 15;20:743. doi: 10.1186/s12864-019-6116-0 (PMC6794883; doi:10.1186/s12864-019-6116-0)

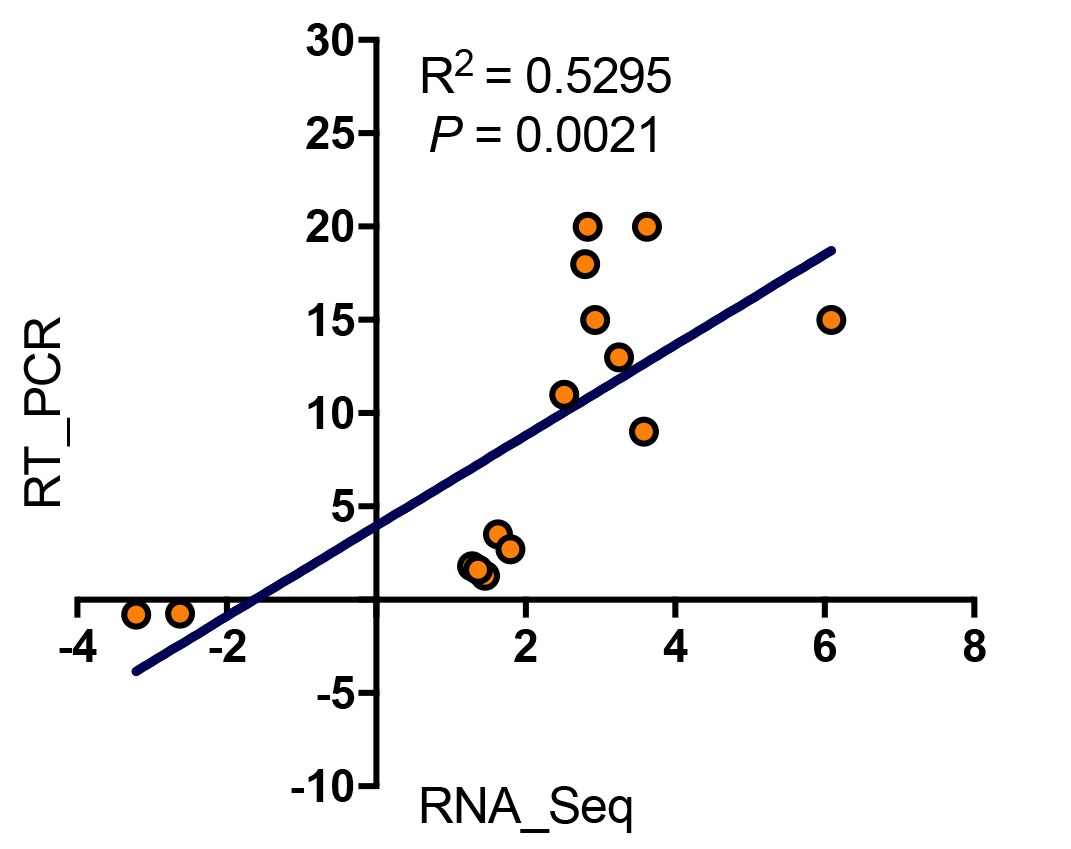

Supplement: Supplementary file 7 — Additional file 7: Figure S1. The correlation analysis between RNA-Seq data and qRT-PCR results [file 12864_2019_6116_MOESM7_ESM.tif]
